# Proteomic Signatures of Antimicrobial Resistance in *Yersinia pestis* and *Francisella tularensis*

**DOI:** 10.3389/fmed.2022.821071

**Published:** 2022-02-10

**Authors:** Brooke L. Deatherage Kaiser, Dawn N. Birdsell, Janine R. Hutchison, Johanna Thelaus, Sarah C. Jenson, Voahangy Andrianaivoarimanana, Mona Byström, Kerstin Myrtennäs, Ryelan F. McDonough, Roxanne D. Nottingham, Jason W. Sahl, Herbert P. Schweizer, Minoarisoa Rajerison, Mats Forsman, David S. Wunschel, David M. Wagner

**Affiliations:** ^1^Pacific Northwest National Laboratory, Chemical and Biological Signatures Group, Richland, WA, United States; ^2^Pathogen and Microbiome Institute, Northern Arizona University, Flagstaff, AZ, United States; ^3^Swedish Defence Research Agency, Chemical, Biological, Radioactive, and Nuclear (CBRN) - Defence and Security, Umeå, Sweden; ^4^Plague Unit, Central Laboratory for Plague, Institut Pasteur de Madagascar, Antananarivo, Madagascar

**Keywords:** proteomics, antimicrobial resistance (AMR), *Yersinia pestis*, *Francisella tularensis*, fatty acid biosynthesis

## Abstract

Antimicrobial resistance (AMR) is a well-recognized, widespread, and growing issue of concern. With increasing incidence of AMR, the ability to respond quickly to infection with or exposure to an AMR pathogen is critical. Approaches that could accurately and more quickly identify whether a pathogen is AMR also are needed to more rapidly respond to existing and emerging biological threats. We examined proteins associated with paired AMR and antimicrobial susceptible (AMS) strains of *Yersinia pestis* and *Francisella tularensis*, causative agents of the diseases plague and tularemia, respectively, to identify whether potential existed to use proteins as signatures of AMR. We found that protein expression was significantly impacted by AMR status. Antimicrobial resistance-conferring proteins were expressed even in the absence of antibiotics in growth media, and the abundance of 10–20% of cellular proteins beyond those that directly confer AMR also were significantly changed in both *Y. pestis* and *F. tularensis*. Most strikingly, the abundance of proteins involved in specific metabolic pathways and biological functions was altered in all AMR strains examined, independent of species, resistance mechanism, and affected cellular antimicrobial target. We have identified features that distinguish between AMR and AMS strains, including a subset of features shared across species with different resistance mechanisms, which suggest shared biological signatures of resistance. These features could form the basis of novel approaches to identify AMR phenotypes in unknown strains.

## Introduction

Antimicrobial resistance (AMR) and multidrug resistance (MDR) represent significant public health threats because antibiotics are the primary therapeutic option for many important pathogens that lack a licensed vaccine (1, 2). To inform effective treatment, it is critical to first determine if the infective strain is AMR. However, most techniques for identifying AMR still rely upon culture, which is time-consuming and resource intensive (3). We sought to address this challenge through discovery of organism signatures that relate to AMR, can be accessed through techniques that do not require culturing the organisms in the presence of multiple different antimicrobials, and could be transitioned to a diagnostic that relies on protein detection such as a lateral flow immunoassay. To do this, we investigated changes in protein expression in AMR strains as compared to genomically-similar antimicrobial susceptible (AMS) isolates of the same pathogens.

Although observation of expressed proteins that directly facilitate AMR (such as the presence of an efflux pump) can identify AMR strains, we explored whether there are additional protein expression changes that go beyond the specific mechanism of resistance in a small, well-defined set of AMR organisms. If AMR acquisition induces a broader impact on the cell that can be observed as changes to cellular physiology, then more global molecular signatures of AMR could be accessible. Acquisition of AMR is generally thought to also bring with it a metabolic cost (4). The mutation of key cellular proteins that are targets of antimicrobials (e.g., DNA gyrase or ribosomal proteins), the acquisition of new AMR determinants via horizontal gene transfer (e.g., inactivating enzymes), or mutational events leading to their expression (e.g., efflux pumps), are thought to change the growth characteristics of organisms. For example, a point mutation of *Campylobacter jejuni gyrA* that confers resistance to fluoroquinolones has been shown to enhance fitness in the animal reservoir (5). By contrast, a point mutation in the *rpsL* gene of *Salmonella typhimurium* that confers streptomycin resistance was shown to negatively impact fitness in culture (6). A more specific example was seen in a multi-drug resistant (MDR) strain of *Stenotrophomonas maltophilia* expressing an efflux pump, which was shown to have distinct metabolic profiles with changes in carbohydrate utilization relative to susceptible strains (7). These examples illustrate that AMR can have measurable impacts on global traits such as bacterial physiology and fitness that might also be observed as changes at the molecular level.

Several studies have focused on transcript and protein changes in response to antimicrobial therapy. At the transcriptome level, MDR strains of *Acinetobacter baumannii* have been shown to have a range of responses to different antimicrobials (8). Multi-drug resistant *A. baumannii* strains had a general increase in expression of genes involved in ATP, RNA, and protein synthesis, and upregulation of TCA cycle, amino acid metabolism, and membrane transport genes. The transcriptomic response of MDR strains of *Serratia marcesens* also showed upregulation of genes encoding membrane transporters (9). Thus, broad changes in gene expression that impact multiple metabolic pathways occur in AMR organisms that encounter antimicrobials during growth.

Proteomic responses to antibiotic treatment in AMR organisms have been investigated in other bacterial pathogens. The proteins known to be directly involved in AMR have been studied, such as *Neisseria gonorrhoeae* efflux pump proteins that were more highly expressed in AMR strains when exposed to azithromycin (10). Specific protein expression changes beyond those proteins directly involved in AMR also have been identified in response to antimicrobial treatment of AMR organisms. Streptomycin and doxycycline induced changes in *Klebsiella pneumoniae* proteins integral to glycolysis, TCA cycle, nitrogen cycle, fatty acid biosynthesis, and reactive oxygen species pathways (11, 12), and proteins involved in AMR *Escherichia coli* DNA repair and fatty acid biosynthesis were also identified (13).

Although these studies are important for improving our understanding of microbial response to antimicrobial exposure by resistant cells, persistent proteomic changes that may be indicative of AMR in the absence of antimicrobial treatment have not been identified. This is an important space to characterize because of the value of identifying the AMR status of infective strains prior to antibiotic treatment. To that end, the goal of this study was to identify whether changes occur in the proteome that accompany AMR as a first step in understanding these proteins as potential signatures of resistance. We have specifically focused on impacted metabolic pathways to identify conserved signatures of AMR that could be useful for clinical detection or in the design of therapeutics that target these changes, as well as to further our understanding of metabolic compensation in AMR strains. We investigated proteomic signatures of AMR *Yersinia pestis* and *Francisella tularensis*, which are the causative agents of the diseases plague and tularemia, respectively (14, 15). We examined proteome profiles of six strains: one MDR *Y. pestis* and two AMR *F. tularensis* strains, plus a closely related AMS strain paired with each of the AMR/MDR strains. We identified protein changes in multiple cellular functions and metabolic pathways, many of which were observed in all three resistant strains, supporting the hypothesis that generalized signatures of AMR may exist.

## Materials and Methods

### Bacterial Strains Utilized in This Study

We utilized a version of MDR *Y. pestis* strain 17/95, referred to in the rest of this manuscript as AMR strain Yp4003, which was isolated from a human in Madagascar in 1995 and is resistant to ampicillin, chloramphenicol, kanamycin, streptomycin, spectinomycin, sulfonamides, tetracycline, and minocycline due to the presence of a ~150 kb self-transmissible plasmid (pIP1202) (16). It is important to note that this list includes almost all of the drugs recommended for plague prophylaxis and therapy, as well as some drugs recommended as alternatives for therapy (17). Yp4003 was paired with AMS strain Yp4181, which is the Yp4003 strain that was cured of pIP1202 following methods described previously (16). In addition, we analyzed virulent AMR isolates from both of the two main subspecies of *F. tularensis*: *F. tularensis* subsp. *tularensis* (Type A) strain FSC016, which is resistant to streptomycin; and *F. tularensis* subsp. *holarctica* (Type B) strain FSC232, which is resistant to rifampicin. Paired AMS isolates from both of the two main subspecies of *F. tularensis* were also examined: *F. tularensis* subsp. *tularensis* (Type A) strain FSC013, and *F. tularensis* subsp. *holarctica* (Type B) strain FSC201. Laboratory work involving all virulent strains was performed in a Biosafety Level 3 (BSL3) laboratory.

### Antibiotic Susceptibility and Growth of *Y. pestis* Strains

The susceptibility/resistance of *Y. pestis* strains (Yp4181 and Yp4003) to antimicrobial agents was confirmed using MIC test strips (bioMe'rieux, Salt Lake, UT) on Mueller-Hinton (MH) agar plates (Fisher Scientific, Lenexa, KS). *Yersinia pestis* bacteria were plated on MH plates to generate lawns and MIC test strips were placed on the lawns. After 48 h of incubation at 28°C, MICs were read according to the manufacturer's instructions. For quality assurance, each batch of plates and MIC test strips was tested using the control *Y. pestis* strain A1122. The breakpoints were interpreted as the lowest concentration yielding no growth by visual inspection.

Growth of the two *Y. pestis* strains was assessed in defined Tryptic Soy broth (TSB) without Dextrose media (Fisher Scientific, Lenexa, KS) at 28–30°C. The MDR strain Yp4003 was grown both in the presence and absence of streptomycin (64 μg/ml). Growth was monitored for 24 h in a flask setup with optical density measurements monitored at time points capturing log and stationary phase growth phases. The experiment was repeated three times with two replicates per strain in each experimental run (Supplementary Figure 1).

### Antibiotic Susceptibility and Growth of *F. tularensis* Strains

The susceptibility of *F. tularensis* strains to antimicrobial agents was determined using MIC test strips (bioMe'rieux) on GCII agar (chocolate agar) containing 1% hemoglobin and 1% IsoVitaleX (14). Bacteria were harvested after incubation for 48 h at 37°C in a 5% CO_2_ atmosphere and suspended in saline solution (0.9% NaCl) at a concentration of 10^8^ colony forming units (cfu)/ml, as determined by optical density measurement at 600 nm (OD_600_). Next, new plates were spread uniformly with 100 μl of the harvest solution and, 10–15 min later, an MIC test strip was placed on each plate. MICs were read after 48 h of incubation at 37°C in a 5% CO_2_ atmosphere according to the manufacturer's instructions. For quality assurance, each batch of plates plus MIC test strips was tested using the control strains *E. coli* ATCC 25922, *Pseudomonas aeruginosa* ATCC 27853, and *Staphylococcus aureus* ATCC 29213. The breakpoints were interpreted in accordance with the EUCAST (18). These standards have not been validated for *F. tularensis* using the MIC test strip method.

Growth of all four *F. tularensis* strains was assessed in defined Chamberlain's media at 37°C. The two AMR strains (FSC016 and FSC232) were grown both in the presence and absence of either streptomycin (64 μg/ml; strain FSC016) or rifampicin (5 μg/ml; strain FSC232). Growth was monitored for 40 h in 96-well plate setup (Tecan) with optical density measurements every second hour using a plate reader (Tecan Infinite^R^ 200 PRO and Magellan). The experiment was repeated three times with four replicates per strain in each experimental run.

### Whole Genome Sequencing and Generation of Protein Annotations

Whole genome sequences were generated for the two *Y. pestis* strains using the Illumina sequencing platform. Genomes were assembled with SPAdes v3.13.1 (19) using default parameters. Contigs were manually removed if they aligned to known contaminants or contained an anomalously low depth of coverage based on raw read mapping with minimap2 v2.1 (20) and depth calculations with Samtools v1.11 (21). The four strains *F. tularensis* were sequenced using both the Illumina and Nanopore platforms. For Illumina sequencing, DNA was extracted using EZ1 extraction robot with EZ1 DNA Investigator Kit (Qiagen GmbH, Germany). For Nanopore sequencing, DNA was extracted using MagAttract HMW DNA kit (Qiagen GmbH, Germany). The Illumina sequencing library was prepared with Nextera XT DNA Library Prep Kit v3 (Illumina Inc., USA) and sequenced on a MiSeq instrument (Illumina Inc., USA) using a MiSeq Reagent Kit v3 (600 cycles). Illumina reads were trimmed using Trimmomatic v.0.35 (22), which removed low quality bases at the end of reads. The nanopore sequencing library was prepared using Ligation Sequencing Kit (SQK-LSK108) with the 1D native barcoding expansion kit EXP-NBD103. The libraries were sequenced for 48 h using a MinION flow cell FLO-MIN106 with R9.4 chemistry (Oxford Nanopore Technologies Ltd., UK). Nanopore reads were basecalled using Albacore v.1.1.1, and adapters were trimmed with Porechop v.0.2.3 (23). Nanopore reads were assembled using Canu v.1.5., and Berokka v.0.1 was used to remove overhang from the assemblies. To correct the Nanopore assemblies, Illumina reads were mapped with bwa-mem v.0.7.17, and the alignment result were used by Pilon v.1.22 for correction. This was done iteratively four times, using the output from pilon as reference for the mapping of Illumina reads. Finally, the assemblies were rotated to start with *dnaA* using Circlator fixstart (24). We confirmed with CanSNPer2 (25) that the pair FSC013/FSC016 are members of A.I.10 subclade and the pair FSC201/FSC232 are members of the B.12 subclade B.22. The annotation of coding regions was performed with Prokka v1.12 (26), utilizing *Yersinia*-specific and *Francisella*-specific annotation databases.

The genome sequences of strains FSC013, FSC016, FSC201, and FSC232 (BioProject PRJNA765161) have been deposited in GenBank/SRA under the accession numbers SAMN16048000/SAMN21545788 [FSC013 (27)], SAMN21545789 (FSC016), SAMN03773972/SAMN21545790 (FSC201), and SAMN21545791 (FSC232). The genome sequences of strains Yp4003 and Yp4181 (BioProject PRJNA765313) have been deposited in GenBank/SRA under the accessions SRR16012151 (Yp4003) and SRR16012150 (Yp4181).

### Sample Collection and Experimental Design

A goal of our study was to ensure that biological replicates and randomization of sample runs were included up front in the experimental design. Including multiple biological replicates helps account for anticipated biological variability, and randomization of sample runs (as well as randomization of sample order during instrument analysis, which was also performed) helps reduce sample collection and run order effects. This is important, as both concerns can confound data analysis.

As described above, two *Y. pestis* and four *F. tularensis* strains were utilized in this study. Each strain was cultured in duplicate flasks on two separate experimental run days in randomized order. Both *Y. pestis* strains were grown in TSB media at 28–30°C and the AMR strain Yp4003 was grown both in the presence (64 μg/ml) and absence of streptomycin. We selected a streptomycin concentration (64 μg/ml) that was well above the Etest breakpoint of *Y. pestis* (4 μg/ml) and well below the highest streptomycin concentration on the MIC test strips (1,024 μg/ml). Early growth phase (mid-exponential; also referred to below as the early time point) samples were collected from the *Y. pestis* strains at 7–10 h post inoculation, whereas late growth phase (stationary; also referred to below as the late time point) samples were collected at 23–24 h post inoculation. All four *F. tularensis* strains were grown in defined Chamberlain's media at 37°C and the two AMR strains (FSC016 and FSC232) were grown both in the presence and absence of either streptomycin (64 μg/ml; strain FSC016) or rifampicin (5 μg/ml; strain FSC232). In the two Type A *F. tularensis* strains (FSC016 and FSC013), early growth phase samples (defined as reaching an OD_600_ value of approximately 0.5) were collected at 14–17 h post inoculation and late growth phase (defined as OD_600_ value of approximately 1.0) samples were collected at 36–38 h post inoculation. The two Type B *F. tularensis* strains (FSC232 and FSC201) grew more slowly, reaching early growth phase (OD_600_ = 0.5) at 23–27 h post inoculation and late growth phase (OD_600_ = 1.0) at 40–48 h post inoculation (Supplementary Figure 2).

Extensive sample processing and sterility testing was conducted prior to shipment of biomass to Pacific Northwest National Laboratory (PNNL) for analysis, as approved by the PNNL Institutional Biological Safety Committee. To collect *Y. pestis* and *F. tularensis* biomass samples, 10 ml of culture broth was pelleted via centrifugation (1,950 x *g* for 10 min) and the supernatant was removed. Pellets were then resuspended in 1 ml 70% ethanol for 20 min at room temperature to achieve 100% inactivation. The inactivated cells were pelleted via centrifugation (20,000 × *g* for 2 min), the ethanol supernatant was removed, and the pellet was resuspended in 1 mL sterile 1x PBS with a pH of 7.4. The cells were washed once in PBS and resuspended in 600 μl 1x PBS solution. Sterility of *Y. pestis* biomass samples was confirmed by plating 10% of the final cell suspension from each sample on a Tryptic Soy Agar (TSA) plate (60 μl), followed by incubation at 28°C for ≥48 h. Sterility of *F. tularensis* biomass samples was confirmed by plating 10% of the final cell suspension from each sample on McLeod agar plates (60 μl), followed by incubation at 37°C for ≥7 days.

### Generation of Protein Data

Biomass samples received at PNNL were thawed, centrifuged at 12,000 x g for 5 min to pellet biomass, the supernatant was removed and discarded, and each sample was resuspended in 200 μl HPLC water. Samples were processed using the MPLEx method (28). Briefly, ice cold 2:1 CHCl_3_:MeOH was added to each sample to yield a 5:1 ratio of (CHCl_3_:MeOH): sample. Samples were vortexed and then centrifuged to separate into upper aqueous and lower organic phases, with a proteinaceous layer separating the upper and lower phases. The aqueous (water-soluble metabolites) and organic (water-insoluble lipids) layers were carefully removed by pipetting and samples were saved in separate fresh tubes for storage at −80°C. The remaining protein layer was pelleted by centrifugation and washed once with ice cold methanol. Protein pellets were resuspended in 100 μl of freshly made lysis buffer (6 M urea, 14.3 mM β-mercaptoethanol in 50 mM Tris-HCl, pH 8) and incubated with shaking (400 rpm) at 60°C for 1 h in a Thermomixer. Following incubation, 900 μl 50 mM ammonium bicarbonate was added to dilute the sample prior to addition of 2 μl Promega Trypsin Gold for protein digestion. Samples were incubated overnight (15–18 h) at 37°C in a Thermomixer with shaking (400 rpm). The next day, samples were removed and peptides were cleaned via solid phase extraction (SPE) with Phenomenex C18-T cartridges according to manufacturer's protocol. Peptide samples were resuspended in 0.1% formic acid, and the Pierce BCA assay was used to determine final peptide concentration. All samples were adjusted to the same peptide concentration (1 μg/μl) prior to LC-MS/MS analysis.

Liquid chromatography separation was performed using an Agilent 1200 HPLC instrument with a 40 cm long 0.15 mm ID fused silica packed with Jupiter 5 μm C-18 resin. Mobile phase A was prepared with 5% acetonitrile and 0.1% formic acid in nano-pure H_2_O; mobile phase B was prepared with 95% acetonitrile and 0.1% formic acid in nano-pure H_2_O. The flow rate was 2 μl per min with a reversed phase gradient transitioning from 0% solution B to 45% solution B over the course of 60 min for separation followed by a wash and regeneration step. An Orbitrap XL mass spectrometer (Thermo Electron, Thousand Oaks, CA) was used to analyze the eluate from the HPLC, which was directly ionized and transferred into the gas phase with an electrospray emitter (operated at 3.5 kV relative to the mass spectrometer interface). The ion transfer tube on the Orbitrap system was maintained at 200°C and 200 V with an ion injection time set for automatic gain control with a maximum injection time of 200 ms for 5 × 10^7^ charges in the linear ion trap. Ion selection was achieved using dynamic parent ion selection in which the five most abundant ions were selected for MS/MS using a 3 m/z window. Each sample was analyzed in technical triplicate.

### Analysis of Protein Data

LC-MS/MS raw data files were searched against the matching genome sequence of each bacterial strain using the search algorithm MaxQuant (29). Parameters used for the search included: 1% protein false discovery rate (FDR), 1% peptide spectrum match FDR, oxidized methionine as a dynamic post-translational modification, matching between runs, and partial tryptic cleavage rules. Within the resulting proteingroups.txt file, the LFQ (normalized) intensities for each protein observed was used for downstream analyses. Normalized protein intensities were imported into the program InfernoRDN (omics.pnl.gov/software/infernordn) for statistical analysis. Intensities were log_2_ transformed and subjected to ANOVA comparisons (for example, datasets from AMR samples vs. datasets from AMS samples). Visualizations such as heat maps and PCA plots were also generated in InfernoRDN. The mass spectrometry proteomics data have been deposited to the ProteomeXchange Consortium via the PRIDE (30) partner repository with the dataset identifier PXD029828.

## Results

### The AMR/AMS *F. tularensis* Strains

*F. tularensis* is naturally resistant to many antibiotic classes *in vitro* (31), including ß-lactams and macrolides for type B major clade B.12 strains (32). The aminoglycosides (streptomycin and gentamicin), the tetracyclines (e.g., doxycycline), and the fluoroquinolones (ciprofloxacin, levofloxacin) are first-line drugs for treatment of tularemia patients (33). Acquired AMR in clinical *F. tularensis* strains has not been reported (14).

The *F. tularensis* Type B AMS/AMR strain pair included in this study (FSC201/FSC232) belongs to subclade B.22 of the major clade B.12; all members of this major clade are naturally resistant to erythromycin [(32) and Table 1]. Strain FSC232 is additionally resistant to rifampicin due to a point mutation in the gene that encodes the beta subunit of RNA polymerase (*rpoB*, C1544T/Ser523Leu, Table 1). FSC232 was paired with AMS *F. tularensis* Type B strain FSC201, which is the parent strain of FSC232 and was isolated from a human in Sweden in 1998. Antimicrobial resistance *F. tularensis* Type A strain FSC016 (subclade A.I.10) is resistant to streptomycin due to a point mutation in *rpsL* (A263G/Lys43Arg, Table 1). FSC016 was paired with AMS *F. tularensis* Type A strain FSC013, which is identical to FSC016 except for the lack of the point mutation in *rpsL*. No cross-resistance was found in FSC016 with other tested aminoglycosides (kanamycin, amakacin, gentamicin, netilmicin, tobramycin) and daptomycin.

**Table 1:** Genome mutations and antimicrobial resistance of *F. tularensis* strains.

**Strain**	**Genome position^a^**	**Gene**	**Amino acid position^b^**	**Amino acid**	**Codon**	**Ery^c^**	**Cip^c^**	**Str^c^**	**Rif^c^**
						**MIC mg/L**	**MIC mg/L**	**MIC mg/L**	**MIC mg/L**
FSC201 (AMS)	316,471	*rpoB*	523	Serine	TCA	>256	0.006	2	0.38
FSC232 (AMR)	316,471	*rpoB*	523	Leucine	TTA	>256	0.004	1.5	>32
FSC013 (AMS)	334,176	*rpsL*	43	Lysine	AAG	0.50	0.008	0.50	0.75
FSC016 (AMR)	334,176	*rpsL*	43	Arginine	AGG	0.38	0.008	>1,024	0.75

a *Position in the FSC201 and FSC013 assembled genomes, respectively*.

b *Position in the FSC201 rpoB gene and FSC013 rpsL gene, respectively*.

c *Ery, erythromycin, Cip, ciprofloxacin, Str, streptomycin, Rif, rifampicin*.

Detailed measurement of the growth rates of the four *F. tularensis* strains in the presence and absence of antibiotics showed a marked fitness cost for FSC232 (Rif^R^) and negligible fitness effect on FSC016 (Str^R^) (Supplementary Figure 2). It is well known that the majority of rifampicin resistance inducing mutations cause a significant fitness cost (34), especially with spontaneous mutations, such as the one found in FSC232 (C1544T/Ser523Leu). Recent studies have emphasized the need of fitness-compensatory mutations for the survival of clinical rifampicin-resistant bacterial strains (35). The observed significant fitness cost of the C1544T indicates that strain FSC232 has not yet accumulated any compensatory mutations to overcome the fitness cost and, confirming this, we identified only a single mutation in strain FSC232 compared to FSC201 via whole genome comparisons—the SNP in *rpoB* conferring rifampicin resistance. As we observe for FSC016 (Str^R^), negligible fitness effect of *rpsL* mutations conferring streptomycin resistance have been reported in other bacteria (36). Mechanistically, it has been proposed that this reflects a failure of these streptomycin mutants to induce stress-inducible sigma factors. In line with this, such mutants grow faster on media with poorer carbon sources compared with rich media (36). Chamberlain's media, as we have used in this study, is a defined minimal liquid media and, accordingly, this might explain the small differences in growth between the AMS strain FSC013 and the FSC016 strain carrying the *rpsL* A263G mutation. Notably, the two type A strains grow faster in Chamberlain's media compared with the two type B strains (Supplementary Figure 2), and higher growth rate of type A strains compared with type B strains also has been reported to occur on solid media (37).

### Overall Protein Expression Is Strongly Influenced by Growth Phase but Only Weakly Influenced by Presence of Antimicrobials

**Figure 1:**
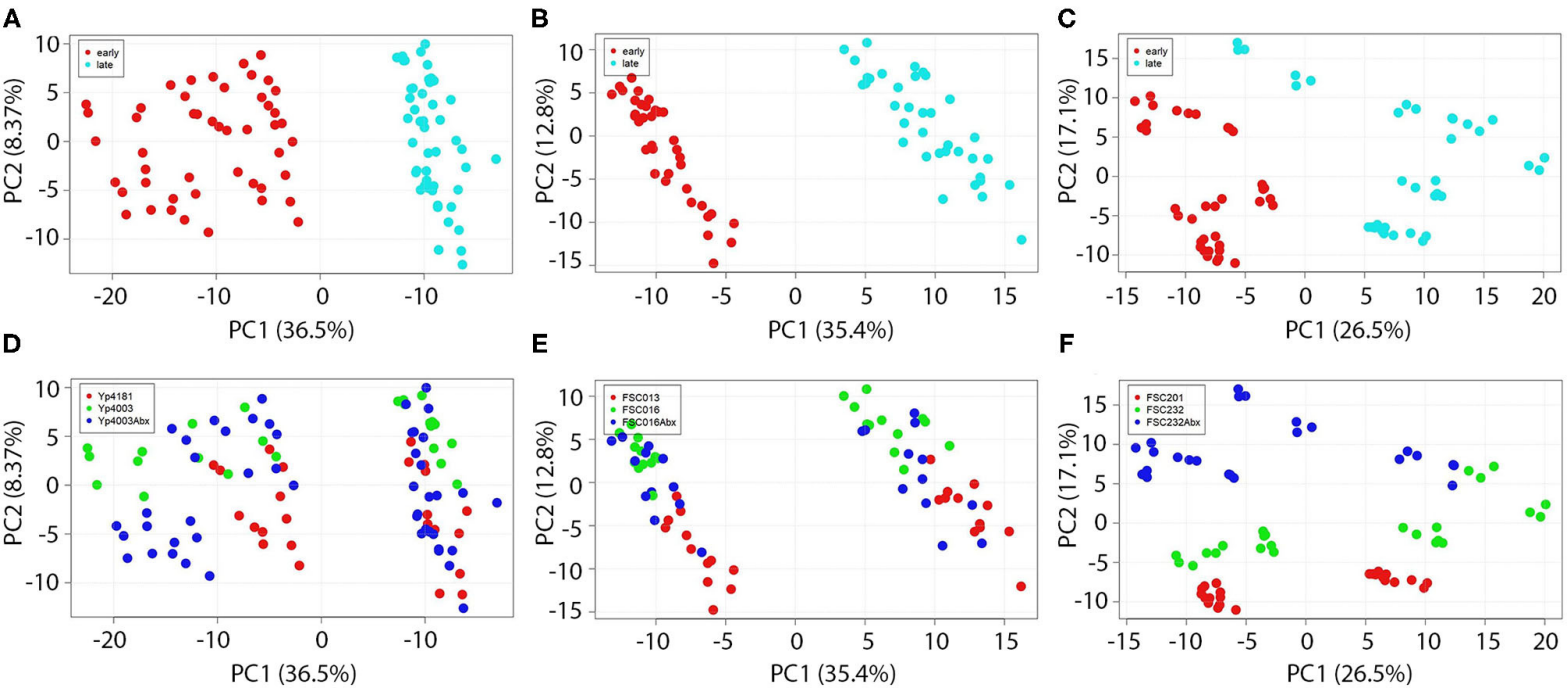
Growth phase significantly influences protein expression, whereas presence of antibiotics produces more subtle effects. Growth phase significantly impacts protein expression in both *Y. pestis* **(A)** and *F. tularensis* **(B,C)**. Proteins observed in biomass sampled at early (mid-log) and late (stationary) growth phases from each AMR/AMS strain pair were subjected to Principal Component Analysis (PCA). The clear separation between samples from early vs. late time points is readily apparent in panels **(A–C)**. Presence of antibiotics in growth media had less significant effects on protein expression **(D–F)**.

Proteomic results demonstrated that the effect of growth phase was significant on protein expression in all three *Y. pestis* and *F. tularensis* strain pairs examined, a phenomenon that also has been observed in other species (38). Figure 1 shows Principal Component Analyses (PCA) of all proteomics data from *Y. pestis* strain pair Yp4181 (AMS) and Yp4003 (AMR, +/– antibiotics; Figures 1A,D), *F. tularensis* strain pair FSC013 (AMS) and FSC016 (AMR, +/– antibiotics; Figures 1B,E), and *F. tularensis* strain pair FSC201 (AMS) and FSC232 (AMR, +/– antibiotics; Figures 1C,F). Figures 1A–C are colored by time point and highlight the significant separation between early and late growth phase samples on PCA axis 1. This demonstrates that protein expression, not surprisingly, is very different in these two phases. Indeed, growth phase was the primary factor accounting for protein expression differences in our study. As such, our subsequent analyses focused on comparisons of data within (not across) either the early time point or the late time point to reduce the conflicting effects of growth phase on other comparisons of more interest (e.g., AMR vs. AMS). Overall, these findings demonstrate that when examining protein expression differences in bacteria as signatures of a specific phenotype (e.g., AMR), or for any other reason, the growth phase that the bacteria were in when samples were collected is important and should be carefully considered.

Presence or absence of antibiotics had a much less pronounced effect on overall cellular protein expression patterns. Protein expression patterns were similar for AMR strains with and without antibiotics present. Figures 1D–F include the same data as in Figures 1A–C but colored by strain identity, including different colors for the AMR strains grown both in the presence and absence of antibiotics. Although there appears to be an effect of the presence of rifampicin on protein expression in AMR *F. tularensis* strain FSC232 along PCA axis 2 (Figure 1F), there is little effect of the presence of streptomycin on protein expression in cultures of AMR *Y. pestis* strain Yp4003 or AMR *F. tularensis* strain FSC016 (Figures 1D,E, respectively). In other words, differences in protein expression between AMR and AMS strains are present even in the absence of antibiotics. This is an important and promising finding because ultimately it would be preferrable to identify the AMR status of an infective strain in an individual before beginning any treatment with antimicrobials so that any information about AMR could be used to guide treatment. As can be visualized in Figures 1D–F, there were differences in protein expression between AMS strains and AMR strains grown in the absence of antibiotics in all three strain pairs but especially in the two *F. tularensis* strain pairs. These differences are discussed in more detail in the following sections. Because it would be ideal to identify signatures of AMR that are present in the absence of antibiotics, we focused most of our subsequent analyses on conditions in which the AMR strains were grown without antimicrobials.

### AMR Status of *F. tularensis* Strains Influences Protein Expression Patterns

Protein expression in *F. tularensis* AMR strains was compared to protein expression of matched AMS strains by *t*-tests. Table 2 outlines the number of proteins found to be significantly differentially expressed in each comparison of interest. We also identified proteins that were expressed in one strain but absent from the comparator strain. The influence of AMR phenotype on protein expression in both examined *F. tularensis* strain pairs was quite significant (Table 2) and more substantial than that observed in the examined *Y. pestis* strain pair (presented below). Expression of between ~20 and 40% of all proteins observed was significantly altered in AMR *F. tularensis* strains harboring resistance against streptomycin (FSC016) or rifampicin (FSC232) compared to their paired AMS strains, and some proteins were only observed in samples of one strain (e.g., expressed by the AMR strain but not the AMS strain). It is interesting to note that these protein differences between AMR and AMS strains are robust across the two strain pairs and also across the two growth phases. The presence of antibiotics does have a small effect on differences in protein expression or presence/absence in AMR strains. However, these differences are small compared to the overall differences observed between AMR and AMS strains.

**Table 2:** Number of proteins differentially expressed or found to differ in presence/absence between two paired sets of AMR and AMS *F. tularensis* strains (*p* < 0.01).

**Comparison**	**Total proteins considered in *t*-tests**	**Proteins with significantly different expression**	**Proteins that differed in presence/absence**
FSC016 vs. FSC013 (early)	558	164	87
FSC016 vs. FSC013 (early + Str)	562	112	90
FSC016 vs. FSC013 (late)	559	166	86
FSC016 vs. FSC013 (late + Str)	560	141	89
FSC232 vs. FSC201 (early)	708	200	124
FSC232 vs. FSC201 (early + Rif)	710	278	122
FSC232 vs. FSC201 (late)	698	220	113
FSC232 vs. FSC201 (late + Rif)	703	247	116

**Figure 2:**
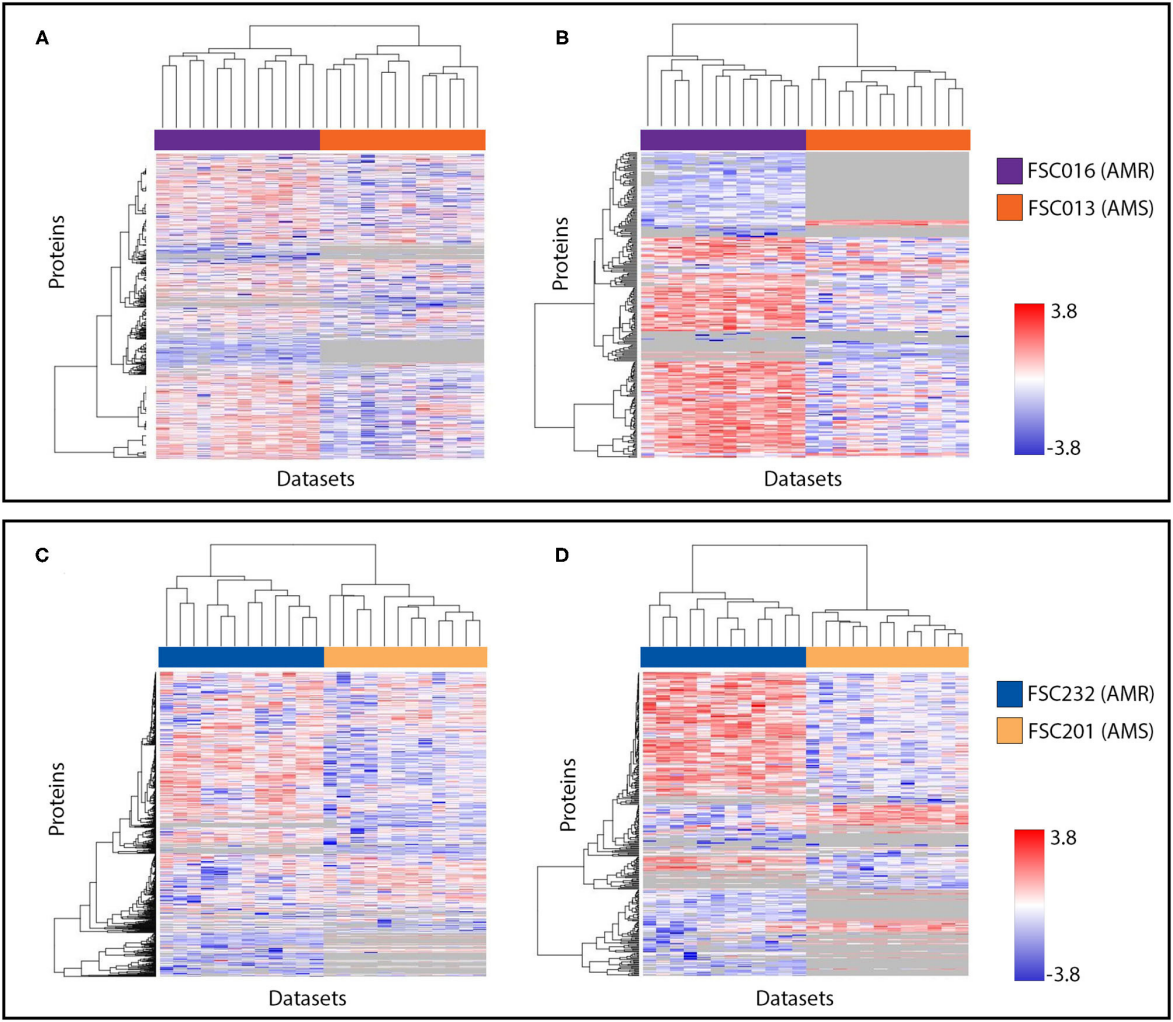
Visualization of protein expression patterns reveals bidirectional protein expression changes in both *F. tularensis* strain pairs. **(A,B)** are data from FSC016/FSC013; **(C,D)** are data from FSC232/FSC201. **(A,C)** include all proteins observed in our analyses of both strain pairs at the early time point. **(B,D)** include the subset of proteins found to be significantly differentially expressed between AMR and AMS strains, as well as those found to be present/absent in this comparison. Proteins are represented in individual rows along the y-axis, and datasets are represented as individual columns along the x-axis. In all heat maps, hierarchical clustering was used to group proteins with similar expression trends. Expression levels are shown on a scale from blue (low) to red (high). Gray indicates the protein was not observed in a dataset.

Protein expression patterns of both *F. tularensis* AMR/AMS strain pairs were also visualized in heat maps. Figure 2 includes data from the early time point without antibiotics present, which is representative of data from either time point. The results are shown for all examined proteins (Figures 1A,C), and proteins with significantly different levels of expression and those present/absent in AMR vs. AMS strains (Figures 1B,D). Note that protein expression changes occurred in both directions, with higher expression in AMR strains in some cases and higher expression in AMS strains in other cases. Importantly, many proteins did not have altered expression patterns in AMR and AMS strains. This is a positive finding, as it indicates that there were no global confounding factors in the experiment that artificially increased intensity values for one sample type over the other. Overall, these findings document that there are many differentially expressed proteins between AMR and AMS strains of *F. tularensis* that can be used as potential signatures of AMR and, again, these potential signatures are present in the absence of antibiotics.

### AMR Status Influences Specific Cell Functions and Specific Pathways in *F. tularensis*

The identity of proteins that are influenced by the AMR phenotype of a strain were of particular interest in this study as we sought to understand any conserved biological changes that may occur in AMR as compared to AMS strains. The subset of proteins found to be significantly different in their expression between the FSC016 (AMR) vs. FSC013 (AMS) and FSC232 (AMR) vs. FSC201 (AMS) strain pairs include proteins in specific metabolic pathways and those that perform multiple cellular functions. Table 3 outlines the major cellular functions/pathways that were most influenced by AMR status, as well as the number of proteins that we classified in each category/pathway. It is worth mentioning that the numbers presented here very likely underrepresent the actual number of proteins in each category as there are many proteins in the genome annotated with unknown or hypothetical functions, which therefore could not be associated with specific pathways or functions. Thus, more proteins may exist that would fit into these categories, but their functions have not yet been determined. This is also true of the *Y. pestis* data presented below.

**Table 3:** Major *F. tularensis* cellular functions and pathways influenced by AMR status.

**Pathways/functions with altered protein abundance between AMR and AMS**	**Directionality**	**FSC232 vs. FSC201**	**FSC016 vs. FSC013**
		***rpoB* mutation (Rif^**R**^)**	***rpsL* mutation (Str^**R**^)**
General lipid metabolism	Up in AMR	8^*^	8
Fatty acid biosynthesis	Up in AMR	8	6
Type 6 secretion system	Down in AMR	14	15
TCA cycle	Up in AMR	9	7
Purine biosynthesis	Up in AMR	9	5
LPS biosynthesis	Up in AMR	11	4
OMPs/OMP processing	Up in AMR	4	6
tRNA ligase	Up in AMR	8	6
Type 4 pili	Up in AMR	1	4

* *Number of proteins in each pathway that were observed to be significantly differently expressed*.

**Figure 3:**
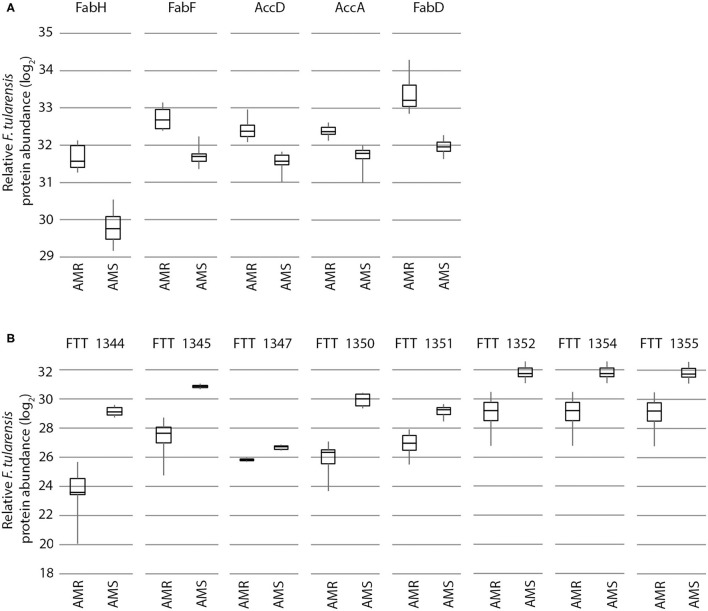
Expression of proteins from specific cellular pathways and functions in *F. tularensis* are influenced by AMR status in a significant, bidirectional manner. Proteins from the fatty acid biosynthesis pathway **(A)** and Type 6 secretion system **(B)** are increased and decreased in the AMR strain vs. the AMS strain, respectively. Shown here is an example with the FSC232 (AMR) and FSC201 (AMS) strain pair (data from the early time point in the absence of antibiotic), but these same trends are also observed in the FSC016/FSC013 strain pair.

Several important patterns can be observed from the data presented in Table 3. First, it is not just individual proteins but rather multiple proteins from each of the listed specific pathways and functions that are differentially expressed between AMR and AMS strains. This indicates that mutations in either *rpoB* or *rpsL* that result in the respective Rif^R^ and Str^R^ *F. tularensis* AMR phenotypes are causing effects on entire pathways and functions as opposed to individual proteins scattered across the proteome. Second, the protein expression differences between *F. tularensis* AMR and AMS strains can be in either direction. Although proteins in most of the listed pathways/functions, such as fatty acid biosynthesis, are upregulated in AMR strains, proteins in the Type 6 secretion system, for example, are downregulated in AMR strains (Figure 3). Third, and most interesting, the observed patterns—including multiple proteins differentially expressed in each pathway/function with upregulation of proteins in some pathways/functions and downregulation in others—is consistent across the two *F. tularensis* AMR/AMS strain pairs. This is despite the two AMR strains having mutations in different cellular targets involved in unrelated biological functions and resulting in resistance to different antibiotics. These observations indicate that there are potential protein expression signatures of AMR that are consistent across the same type of resistance mechanism, irrespective of the cellular target. This is important as global signatures of AMR consistent across different affected cellular functions could be useful across a wide diversity of pathogens and may indicate common biological compensation mechanisms that occur regardless of the mechanism of resistance.

### Protein Expression Changes Occur Between AMR and AMS *Y. pestis* Strains

Protein expression of the *Y. pestis* AMR strain Yp4003 was compared to protein expression of the matched AMS strain Yp4181 by *t*-test. Table 4 outlines the total proteins considered as well as the number of proteins found to be significantly differently expressed in each comparison of interest. We also identified proteins that were expressed in one strain but absent from the comparator strain. Overall, many proteins demonstrated altered expression patterns between the AMR and AMS *Y. pestis* strains, although there were fewer differences than that observed in the two *F. tularensis* strain pairs (Table 3). Interestingly, growing the AMR *Y. pestis* strain in the presence of streptomycin resulted in fewer significant differences in expressed proteins between the AMR strain and the AMS strain compared to growing the AMR strain in the absence of streptomycin. This same pattern was observed in our experiments with the *F. tularensis* AMR/AMS strain pair (FSC016/FSC013) that also involved streptomycin, but the opposite pattern was observed in our experiments with the *F. tularensis* AMR/AMS strain pair (FSC232/FSC201) that differed by rifampicin resistance (Table 3).

**Table 4:** Number of proteins differentially expressed or found to differ in presence/absence between AMR and AMS *Y. pestis* strains (*p* < 0.01).

**Comparison**	**Total proteins considered in *t*-tests**	**Proteins with significantly different expression**	**Proteins that differed in presence/ absence**
Yp4003 vs. Yp4181 (early)	885	159	24
Yp4003 vs. Yp4181 (early + Str)	827	66	26
Yp4003 vs. Yp4181 (late)	883	140	19
Yp4003 vs. Yp4181 (late + Str)	884	62	20

**Figure 4:**
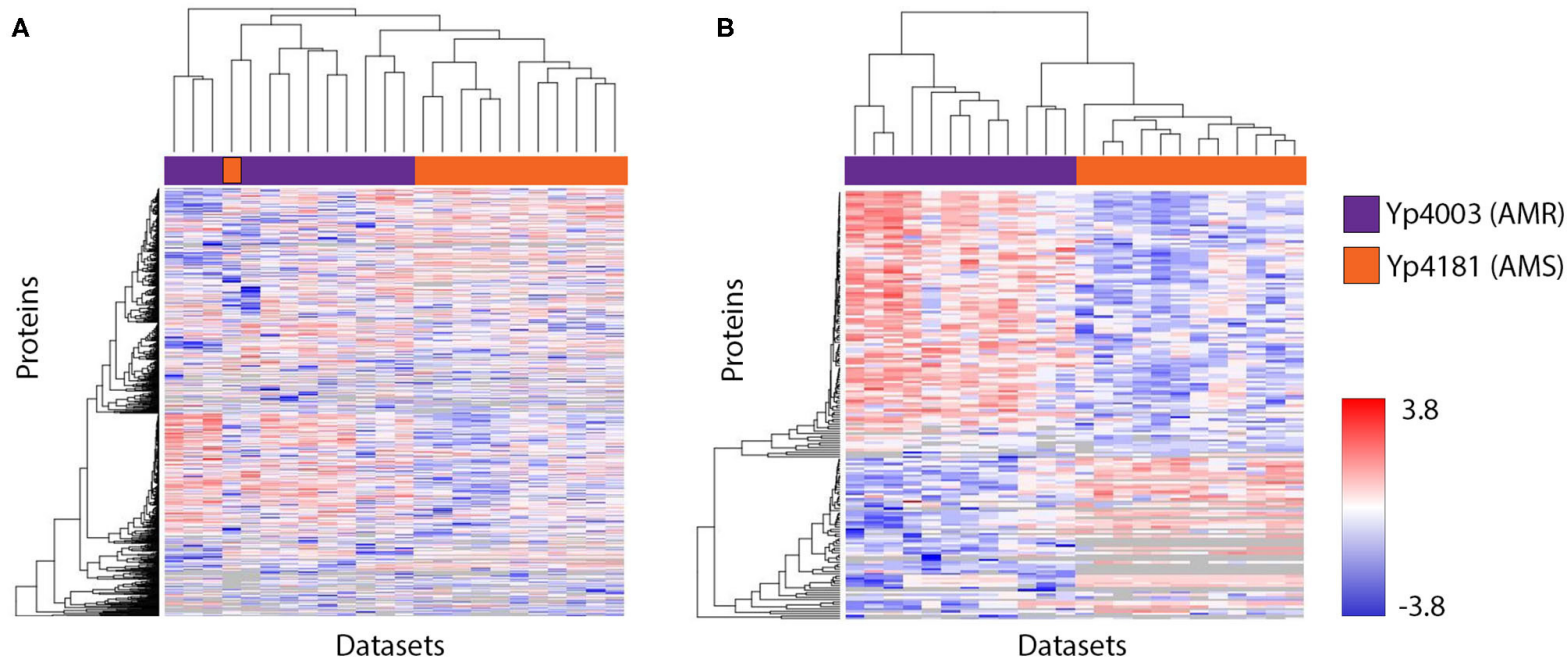
Visualization of protein expression patterns shows bidirectional protein expression changes in *Y. pestis* strain pair Yp4003/Yp4181. **(A)** includes all proteins observed in our analyses at the early time point (no streptomycin). **(B)** includes the subset of proteins found to be significantly differentially expressed between AMR and AMS strains, as well as those found to be present/absent in this comparison. Proteins are represented in individual rows along the y-axis, and datasets are represented as individual columns along the x-axis. In all heat maps, hierarchical clustering was used to group proteins with similar expression trends. Expression levels are shown on a scale from blue (low) to red (high). Gray indicates the protein was not observed in a dataset.

Protein expression patterns of the *Y. pestis* AMR/AMS strain pair were also visualized in heat maps. Figure 4 includes data from the early time point without streptomycin present. The results are shown for all examined proteins (Figure 4A), and proteins with significantly different levels of expression and those present/absent in AMR vs. AMS strains (Figure 4B). As was observed for *F. tularensis* (Figure 2), protein expression changes in *Y. pestis* occurred in both directions, with higher expression in the AMR strain in some cases and higher expression in the AMS strain in other cases. However, there were fewer examples of *Y. pestis* proteins that were present in one condition and absent from the other. Importantly, we also observed proteins for which expression was unaltered. Again, this is a positive finding as it indicates that there were not global confounding factors in the experiment that artificially increased intensity values for one sample type over the other. Overall, these findings document that, similar to observations with *F. tularensis*, there are many differentially expressed proteins between the AMR and AMS strains of *Y. pestis* that can be used as potential signatures of AMR and, again, these potential signatures are present in the absence of antibiotics.

**Figure 5:**
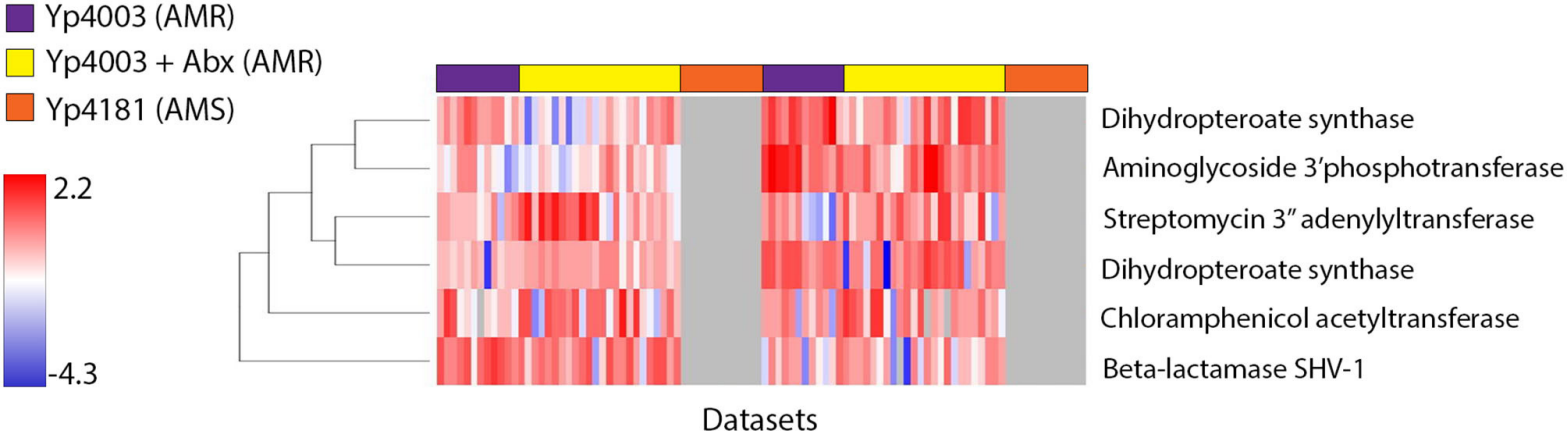
Expression of AMR-conferring proteins encoded by the *Y. pestis* MDR plasmid pIP1202 occurs both in the presence and absence of streptomycin and at both early and late time points. Proteins are represented in individual rows along the y-axis, and datasets are represented as individual columns along the x-axis. Datasets from *Y. pestis* AMR strain Yp4003 grown without streptomycin are highlighted across the top of the image in purple, datasets of Yp4003 grown with streptomycin are highlighted in yellow, and datasets of *Y. pestis* AMS strain Yp4181, which does not contain the MDR plasmid, are highlighted in orange. Hierarchical clustering was used to group proteins with similar expression trends. Expression levels are shown on a scale from blue (low) to red (high). Gray indicates the protein was not observed in a dataset.

As described above, AMR in the two *F. tularensis* AMR strains that we examined is due to two different point mutations. However, resistance in the *Y. pestis* AMR strain (Yp4003) is due to the presence of a self-transmissible MDR plasmid (39) that, to date, has not been reported from any other *Y. pestis* strain (40). This 183 kb MDR plasmid contains hundreds of genes (39), including many genes associated with resistance to a number of different antibiotics. We examined expression of AMR-conferring proteins encoded by the MDR plasmid, both in the presence and absence of streptomycin. Figure 5 shows a heat map of expression patterns of proteins encoded by AMR-related genes on the MDR plasmid in the *Y. pestis* AMR strain (Yp4003) with and without streptomycin present during growth, as well as the paired AMS strain (Yp4181) that has been cured of the MDR plasmid. In all, six proteins responsible for AMR to one or more antimicrobials were expressed in the presence and/or absence of streptomycin, and at both early and late time points. Several interesting patterns can be observed from these data. First, as expected, there was no expression of proteins from the MDR plasmid in the AMS strain (Yp4181) as the genes that encode these proteins are not present in this strain. Second, a number of these AMR-associated proteins appear to be constitutively expressed as they are expressed by AMR strain Yp4003 both in the presence and absence of streptomycin. Together, these two patterns indicate that there are multiple proteins encoded on the MDR plasmid that could be used as potential signatures for the presence of this plasmid even in the absence of antibiotics. A third pattern that can be observed in Figure 5 is that some of the observed AMR-associated proteins were differentially expressed when AMR strain Yp4003 was grown in the presence and absence of streptomycin. Table 5 lists the six AMR-conferring proteins encoded by the MDR plasmid that were observed in our study; we performed *t*-tests to determine whether the presence of streptomycin significantly influenced expression of these proteins at each time point. Aminoglycoside 3″-adenylyltransferase, which is associated with streptomycin-resistance, is significantly upregulated in the presence of streptomycin, both at the early and late time points. Expression of three other proteins was also impacted at one (aminoglycoside 3′-phosphotransferase; dihydropteroate synthase Sul1 Yp4003_04286) or both (dihydropteroate synthase Sul2 Yp4003_00191) time points by the presence of streptomycin. However, expression of beta-lactamase SHV-1 and chloramphenicol acetyltransferase did not significantly change with the presence of streptomycin. The protein(s) responsible for resistance to tetracycline and minocycline were not observed in our data even though multiple genes that encode these proteins are present on the MDR plasmid. However, this was not unexpected because our samples were not grown in the presence of these drugs, which are likely required for induction of expression of genes for proteins conferring resistance to tetracyclines.

**Table 5:** Antimicrobial resistance-conferring proteins encoded on the *Y. pestis* MDR plasmid pIP1202 that were observed in this study and their differential expression in the presence of streptomycin.

**Locus ID**	**Protein description**	**Confers resistance to:**	**Differential expression when streptomycin was present in the growth media?**	
			**Early**	**Late**
YpIP275_pIP1202_0175	Beta-lactamase SHV-1	Ampicillin	No	No
YpIP275_pIP1202_0063	Chloramphenicol acetyltransferase	Chloramphenicol	No	No
YpIP275_pIP1202_0055	Streptomycin resistance protein A	Kanamycin	No	Yes
YpIP275_pIP1202_0190	Aminoglycoside 3”-adenylyltransferase	Streptomycin and spectinomycin	Yes	Yes
YpIP275_pIP1202_0188	Dihydropteroate synthase (Sul1)	Sulfamethoxazole	Yes	No
YpIP275_pIP1202_0073	Dihydropteroate synthase (Sul2)	Sulfamethoxazole	Yes	Yes

### AMR Status Influences Similar Cell Functions and Pathways in Both *Y. pestis* and *F. tularensis*

**Figure 6:**
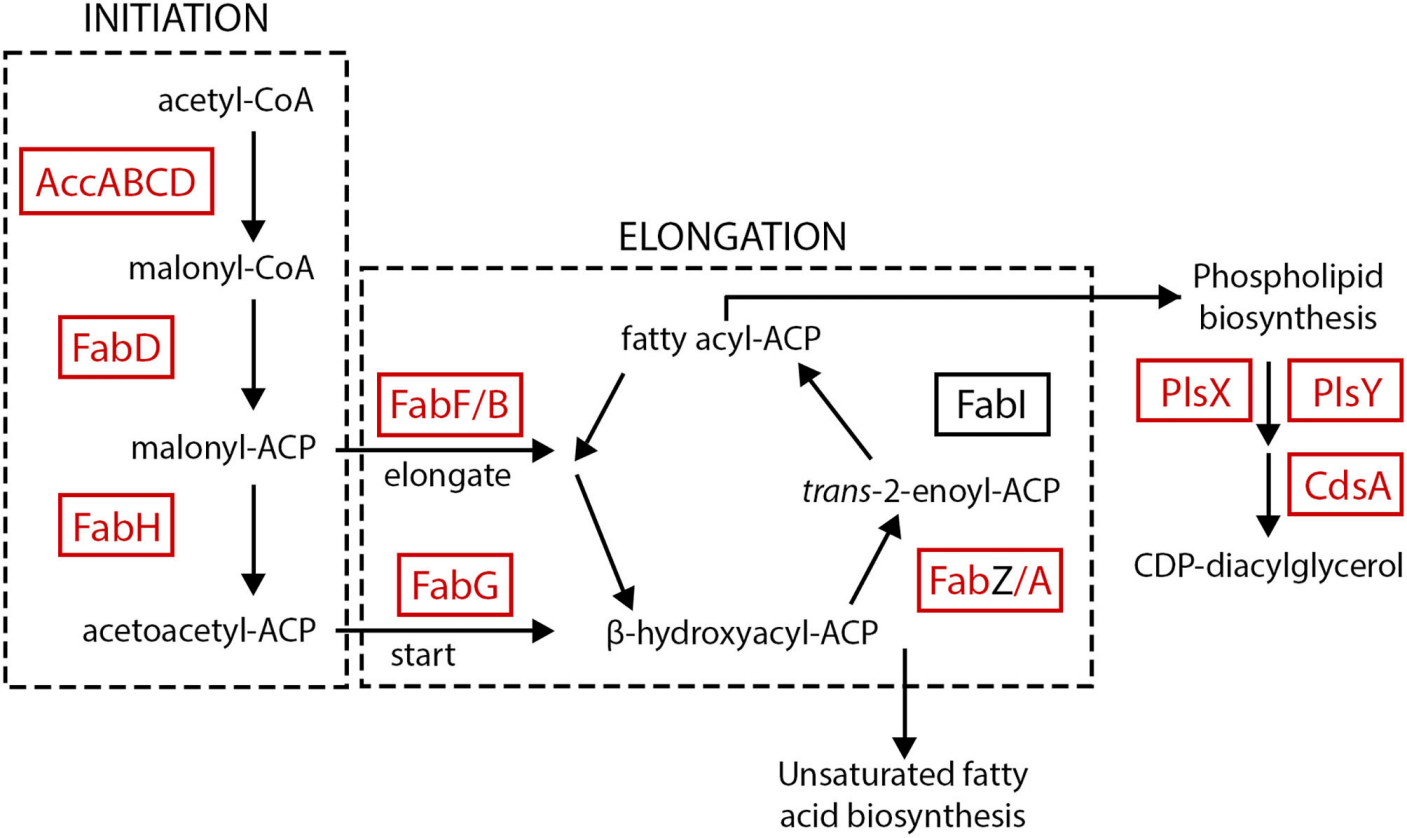
Expression of proteins in the fatty acid biosynthesis pathway. Almost all of the proteins in the fatty acid biosynthesis pathway were increased in AMR strains of both *F. tularensis* AMR/AMS strain pairs and the *Y. pestis* AMR/AMS strain pair. Proteins increased in *Y. pestis* and/or *F. tularensis* AMR strains are indicated by red text and boxes.

Despite being different species and having very different mechanisms of AMR (i.e., target mutations in *F. tularensis* and enzyme mediated resistance in *Y. pestis*), many of the same general metabolic pathways and cellular functions that were influenced by AMR status in *F. tularensis* are also influenced by AMR status in *Y. pestis*. Table 6 outlines the major cellular functions/pathways that were most influenced by AMR status in both *Y. pestis* and *F. tularensis*, as well as the number of proteins that we classified in each category/pathway. Note that multiple proteins from each of the listed specific pathways and functions are differentially expressed between AMR and AMS strains of both strain pairs of *F. tularensis* and also the *Y. pestis* strain pair. In some cases, we have observed differential expression of nearly all of the proteins in a given metabolic pathway, such as the fatty acid biosynthesis pathway (Figure 6). And, with the exception of the purine biosynthesis pathway, the directionality of the protein expression differences associated with these pathways and functions are also similar across both *F. tularensis* and *Y. pestis*. This documents that there are potential protein expression signatures of AMR that are consistent across two different mechanisms of resistance and multiple species, providing more evidence that there may be global signatures of AMR.

**Table 6:** Major *Y. pestis* cellular functions and pathways influenced by AMR status (and compared to those observed for *F. tularensis*).

**Pathways/functions with altered protein abundance between AMR and AMS**	**Directionality**	**FSC232 vs. FSC201**	**FSC016 vs. FSC013**	**Yp4003 vs. Yp4181**
		***rpoB* mutation (Rif^**R**^)**	***rpsL* mutation (Str^**R**^)**	**MDR plasmid**
General lipid metabolism	Up in AMR	8^*^	8	–
Fatty acid biosynthesis	Up in AMR	8	6	6
Type 6 secretion system	Down in AMR	14	15	4
TCA cycle	Up in AMR	9	7	5
Purine biosynthesis	*Ft*: Up in AMR *Yp*: Down in AMR	9	5	13
LPS biosynthesis	Up in AMR	11	4	8
OMPs/OMP processing	Up in AMR	4	6	12
tRNA ligase	Up in AMR	8	6	9
Type 4 pili	Up in AMR	1	4	–

* *Number of proteins in each pathway that were observed to be significantly differently expressed*.

## Discussion

We have identified and characterized multiple protein expression patterns that differentiate AMR and AMS strains of both *F. tularensis* and *Y. pestis*. These potential protein signatures of AMR are present even when the organisms were grown in the absence of antibiotics. This is not too surprising since many AMR determinants are constitutively expressed. In addition, the protein expression features were present in organisms of different genera and species that harbor two very different mechanisms of AMR. These results support the hypothesis that conserved phenotypic features of AMR exist and can be detected in the proteome. Although only one of the AMR/MDR strains exhibited *in vitro* growth defects as compared to the paired AMS strains in this study, there were still identifiable metabolic changes irrespective of presence or absence of antibiotics during growth. These seemingly conserved metabolic changes are reflected in the proteome, and further study with additional organisms and resistance mechanisms will indicate whether these changes are universal signatures of AMR.

## Data Availability Statement

The original contributions presented in the study are publicly available. This data can be found here: Sequence Read Archive (SRA): SRR16012151 (Yp4003), SRR16012150 (Yp4181); BioSample Database: SAMN21545788 (FSC013), SAMN21545789 (FSC016), SAMN21545790 (FSC201), SAMN21545791 (FSC232); PRIDE Database: PXD029828.

## Author Contributions

BDK, DB, JH, JT, VA, MB, MR, MF, DSW, and DMW designed the experiments. BDK, DB, JH, JT, VA, MB, RM, RN, and MR performed the experiments. KM and JS performed genome sequencing and analysis. BDK and SJ performed proteomic data analysis. BDK, DSW, DMW, DB, and MF drafted the manuscript. All authors contributed to data interpretation, as well as writing, critically reviewing, revising, and approving the final manuscript for submission.

## Funding

This work was funded by award HDTRA1-16-1-0052 from the United States Defense Threat Reduction Agency (DTRA).

## Conflict of Interest

The authors declare that the research was conducted in the absence of any commercial or financial relationships that could be construed as a potential conflict of interest.

## Publisher's Note

All claims expressed in this article are solely those of the authors and do not necessarily represent those of their affiliated organizations, or those of the publisher, the editors and the reviewers. Any product that may be evaluated in this article, or claim that may be made by its manufacturer, is not guaranteed or endorsed by the publisher.

## Supplementary Material

Supplementary Figure 1 Growth of *Y. pestis* strains in the presence and absence of antibiotics. Growth was assessed by optical density measurements during two growth phase windows (log phase between 9 and 12 h post-inoculation and stationary phase growth at 24 h post inoculation) for *Y. pestis* Yp4003 (AMR) and Yp4181 (AMS). Yp4003 was grown in the presence and absence of streptomycin (64 μg/ml).

Supplementary Figure 2 Growth of *F. tularensis* subsp. *tularensis* (Type A) and subsp. *holarctica (*Type B) AMR/AMS strains in Chamberlain's media at 37°C. Growth was assessed by optical density measurements over 40 h. The two AMR strains (FSC016 Type A and FSC232 Type B), were grown both in the presence and absence of either streptomycin (64 μg/ml; strain FSC016) or rifampicin (5 μg/ml; strain FSC232). The growth rate of the Type A strains FSC013 AMS and FSC016 AMR were relatively higher compared to the Type B strains FSC201 AMS and FSC232 AMR. Plotted values represent means and standard deviation from measurement of four replicates in three experimental runs (*n* = 12).
