# Supplementary figures and images for: Proteomic Signatures of Antimicrobial Resistance in Yersinia pestis and Francisella tularensis
Source: Front Med (Lausanne). 2022 Feb 10;9:821071. doi: 10.3389/fmed.2022.821071 (PMC8866660; doi:10.3389/fmed.2022.821071)

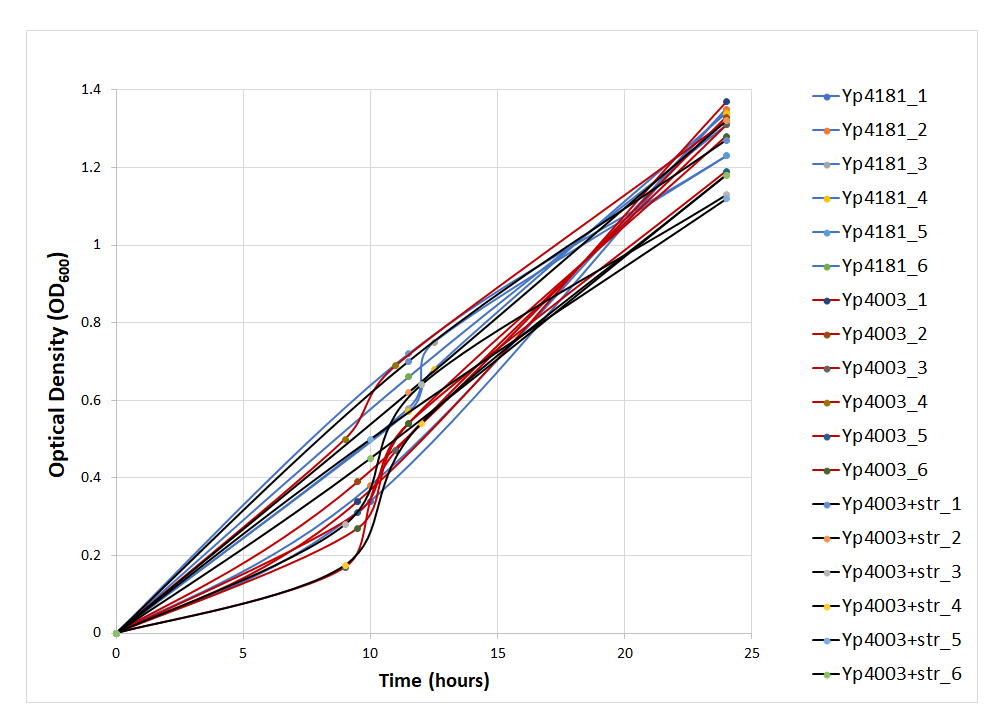

Supplement: Supplementary Figure 1 — Growth of Y. pestis strains in the presence and absence of antibiotics. Growth was assessed by optical density measurements during two growth phase windows (log phase between 9 and 12 h post-inoculation and stationary phase growth at 24 h post inoculation) for Y. pestis Yp4003 (AMR) and Yp4181 (AMS). Yp4003 was grown in the presence and absence of streptomycin (64 μg/ml). [file Image_1.TIF]

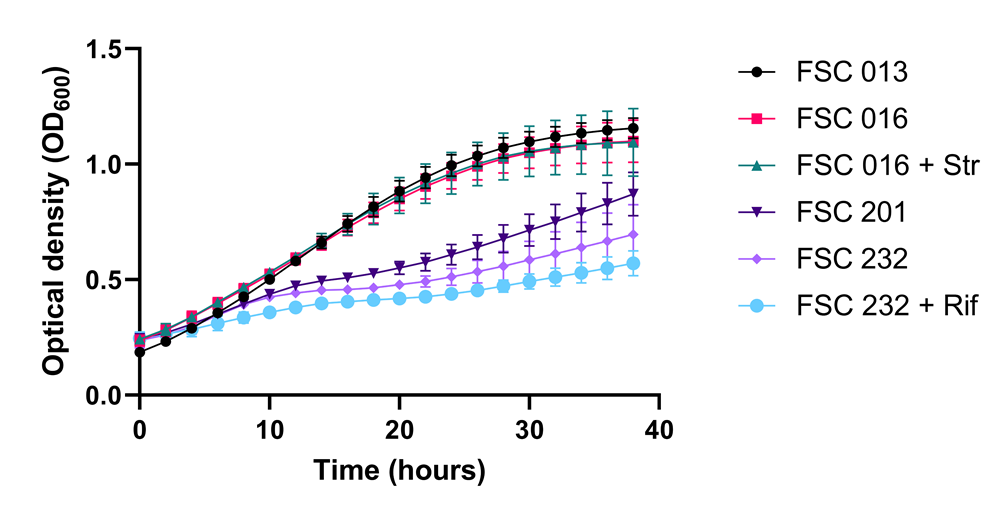

Supplement: Supplementary Figure 2 — Growth of F. tularensis subsp. tularensis (Type A) and subsp. holarctica (Type B) AMR/AMS strains in Chamberlain's media at 37°C. Growth was assessed by optical density measurements over 40 h. The two AMR strains (FSC016 Type A and FSC232 Type B), were grown both in the presence and absence of either streptomycin (64 μg/ml; strain FSC016) or rifampicin (5 μg/ml; strain FSC232). The growth rate of the Type A strains FSC013 AMS and FSC016 AMR were relatively higher compared to the Type B strains FSC201 AMS and FSC232 AMR. Plotted values represent means and standard deviation from measurement of four replicates in three experimental runs (n = 12). [file Image_2.TIF]
